# Advancing Physical Therapy: Integrating Biomechanics, Neuroscience, and Systemic Health

**DOI:** 10.3390/medicina62081585

**Published:** 2026-08-18

**Authors:** Manuel González-Sánchez, Laura Zlibinaite

**Affiliations:** 1 Department of Physiotherapy, Faculty of Health Sciences, University of Málaga, 29071 Málaga, Spain; 2 Institute of Biomedicine of Málaga (IBIMA), 29010 Málaga, Spain; 3 Department of Rehabilitation, Kauno Kolegija Higher Education Institution, Muitines 15, LT-44280 Kaunas, Lithuania

Physical therapy has undergone substantial evolution in recent decades, extending beyond post-surgical and post-traumatic rehabilitation to encompass prevention, health promotion, differential screening, and the management of complex movement-related conditions. This evolution reflects the profession’s commitment to optimizing human movement, improving patient-centered outcomes, and advancing evidence-informed practice. Contemporary physical therapy increasingly integrates diagnostic reasoning, preventive strategies, biopsychosocially informed interventions, and rehabilitation technologies. Recent syntheses support biopsychosocial approaches [1,2] and describe a rapidly expanding landscape of wearable, digital, and artificial intelligence-supported rehabilitation, while emphasizing the need for stronger clinical and economic evaluation before widespread implementation [3,4,5,6].

This Special Issue of the journal *Medicina*, entitled “Physical Therapy: A New Perspective”, aims to explore, analyze, and disseminate the most recent scientific advancements, innovative methodologies, and emerging paradigms across the field of physical therapy. By compiling eight rigorous and diverse research papers, this issue offers a comprehensive platform for researchers, clinicians, and academic leaders to share interdisciplinary insights that redefine contemporary rehabilitation practice, spanning neurophysiology, biomechanics, metabolic health, and clinical reasoning.

A primary focus within this volume involves understanding motor control and neural adaptation following joint pathology. Tedeschi et al. [7] address arthrogenic muscle inhibition (AMI) in individuals with chronic ankle instability (CAI), a condition associated with persistent functional limitations. AMI constitutes an important neurophysiological barrier characterized by involuntary neural inhibition that can restrict voluntary muscle activation. Through a narrative review of five studies, the authors report that peripheral strengthening alone may not fully address spinal and cortical alterations. The available evidence suggests potential benefits from multimodal strategies combining manual therapy, sensorimotor exercise, and cortical neuromodulation via transcranial direct current stimulation (tDCS). However, methodological heterogeneity and short-term follow-up limit generalizability; these approaches should therefore be regarded as promising rather than definitively superior to isolated interventions.

Biomechanical adaptation and core stability in unique athletic populations also represent a relevant avenue of investigation. Ilhan and Erbahceci [8] evaluated competitive amputee soccer players in the Turkish Super League, a population exposed to distinctive biomechanical demands associated with single-leg locomotion and forearm-crutch use. Using pressure biofeedback units, isokinetic dynamometry, and functional performance tests, the researchers explored associations between physiological characteristics and athletic performance. Core stabilization capacity explained 86.1% of the variance in single-leg triple-hop distance and was strongly associated with 10 m sprint performance. Given the cross-sectional design, these findings indicate association rather than causation. They suggest that lumbopelvic control and trunk endurance may be relevant contributors to functional performance in amputee soccer players, but they do not establish that these factors are primary determinants or that they surpass the influence of knee muscle strength.

The systemic physiological implications of common physical therapy exercises are further examined by Yu et al. [9], who conducted a randomized crossover study comparing hemodynamic responses to Valsalva maneuver and abdominal bracing in healthy young adults. Although both techniques were found to increase intra-cavity pressure, their vascular responses differed. The Valsalva maneuver increased the carotid artery pulsatility index, reflecting greater central vascular resistance, whereas abdominal bracing reduced peripheral oxygen saturation and increased peripheral heart–finger pulse wave velocity. Cerebral oxygen saturation decreased during both maneuvers. Because the study was conducted in a small sample of healthy young adults, the findings are best interpreted as mechanistic and hypothesis-generating rather than as direct clinical guidance for patients with cardiovascular disease.

Extending the scope of physical therapy into geriatric and metabolic health, Gökçelik et al. [10] investigated the effects of a four-week progressive inspiratory muscle training (IMT) protocol in healthy older women. Using threshold inspiratory devices calibrated to 40% of maximal inspiratory pressure, the authors observed increases in diaphragm thickness (11.44%) and abdominal wall muscle thickness (up to 12.7%), together with changes in fatty liver density on computed tomography. These results suggest that targeted respiratory training may influence respiratory and abdominal muscle morphology and potentially affect metabolic imaging markers. Nevertheless, the short intervention period and the specific study population mean that effects on sarcopenia or hepatic steatosis require confirmation in larger, longer-term clinical trials.

In a related metabolic investigation using an animal model of obesity, Akbulut et al. [11] examined the combined regulatory effects of aerobic exercise and metformin administration. Obesity disrupts several metabolic signaling molecules involved in energy homeostasis. In obese rats, the combination of exercise and metformin modulated biomarkers including irisin, adropin, copeptin, adiponutrin, and serum uric acid. These preclinical findings suggest a potential interaction between exercise and pharmacological treatment in the regulation of cardiometabolic pathways; however, their translation to human physical therapy and rehabilitation requires appropriately designed clinical studies.

Addressing clinical reasoning, patient safety, and primary care screening, Storari et al. [12] conducted a systematic review examining how “red flags” are defined and operationalized across musculoskeletal clinical practice guidelines. Red flags are key clinical indicators that alert practitioners to potential serious underlying pathologies, such as malignancies, fractures, or infections. The review exposed a critical lack of standardized definitions and consensus across existing guidelines, leading to clinical ambiguity, false positives, and practice variability. Given the global expansion of direct-access physical therapy, the authors emphasize the urgent need for a unified operational definition and advocate for evaluating red flags in diagnostic “clusters” rather than isolated signs to optimize triage and medical referral accuracy.

The psychological and educational dimensions of chronic pain management are explored by Péter et al. [13], who surveyed healthcare professionals regarding their knowledge of pain neurophysiology, beliefs about chronic low back pain, and levels of kinesiophobia. Their findings showed that a substantial proportion of practitioners continued to endorse traditional biomedical beliefs linking pain closely to structural damage and disability. The authors also observed a significant negative association between knowledge of pain neurophysiology and kinesiophobia: clinicians with greater knowledge reported less fear of movement. Although the cross-sectional design does not establish causality, the findings support strengthening pain neuroscience education and biopsychosocial clinical reasoning in undergraduate and continuing professional education, aiming to reduce the transmission of maladaptive fear-avoidance beliefs to patients. Recent meta-analytic evidence also indicates that pain neuroscience education added to exercise can improve pain and disability in chronic spinal pain, although optimal delivery and generalizability across patient populations require further study [14].

Finally, Brognara et al. [15] bridged movement analysis and clinical biochemistry by examining postural instability in patients with diabetic foot. Using wearable inertial sensors and salivary biomarker assays, the researchers identified statistically significant associations between salivary advanced glycation end-products (AGEs) and measures of postural sway. These exploratory findings suggest that salivary AGEs may have potential as adjunctive, non-invasive markers of postural instability. Prospective validation, assessment of diagnostic accuracy, and evaluation of clinical utility are required before these biomarkers can be used to predict risk of fall or guide fall-prevention strategies.

Collectively, the contributions to this Special Issue illustrate the broadening scope and multisystemic relevance of modern physical therapy. From cortical neuromodulation and biomarker research to clinical reasoning and metabolic exercise interventions, the studies highlight important opportunities for interdisciplinary, evidence-informed care. At the same time, the evidence spans narrative reviews, cross-sectional and exploratory studies, preclinical research, and relatively small clinical trials; conclusions should therefore be interpreted in accordance with the design and limitations of each study. Moving forward, the physical therapy community should prioritize standardized diagnostic definitions, adequately powered controlled and longitudinal trials, external validation of emerging biomarkers, implementation of biopsychosocial practice, and rigorous clinical, technological, and economic assessment of rehabilitation innovations [1,2,3,4,5,6,14]. We hope this Special Issue serves as a valuable resource that inspires innovation, fosters interdisciplinary collaboration, and contributes to higher-quality care for patients worldwide.
